# Crystal structure of diethyl [(4-nitro­phenyl­amino)(2-hy­droxy­phen­yl)meth­yl]phospho­nate methanol monosolvate

**DOI:** 10.1107/S1600536814018649

**Published:** 2014-08-30

**Authors:** QingMing Wang, Feng Su, TingTing Yang, LiPing Lu, MiaoLi Zhu

**Affiliations:** a School of Pharmacy, Yancheng Teachers’ University, Yancheng, Jiangsu 224051, People’s Republic of China; b Institute of Molecular Science, Key Laboratory of Chemical Biology and Molecular Engineering of the Education Ministry, Shanxi University, Taiyuan, Shanxi 030006, People’s Republic of China

**Keywords:** crystal structure, α-amino­phospho­nic acids, phospho­nate salts, hydrogen bonding

## Abstract

In the title compound, C_17_H_21_N_2_O_6_P·CH_3_OH, the planes of the 4-nitro­aniline and 2-hy­droxy­phenyl groups form a dihedral angle of 84.04 (8)°. The P atom exhibits tetra­hedral geometry involving two *O*-ethyl groups, a Cα atom and a double-bonded O atom. In the crystal, O—H⋯O, N—H⋯O and C—H⋯O hydrogen bonds link the α-amino­phospho­nic acid and methanol mol­ecules into chains that propagate parallel to the *a* axis.

## Related literature   

For background to the synthesis and properties of α-amino­phospho­nic acids, see: Allen *et al.* (1978 ▶); Arizpe *et al.* (2011 ▶); Cherkasov & Galkin (1998 ▶); Sieńczyk & Oleksyszyn (2009 ▶). For structures of related compounds, see: Li *et al.* (2008 ▶); Wang *et al.* (2012 ▶).
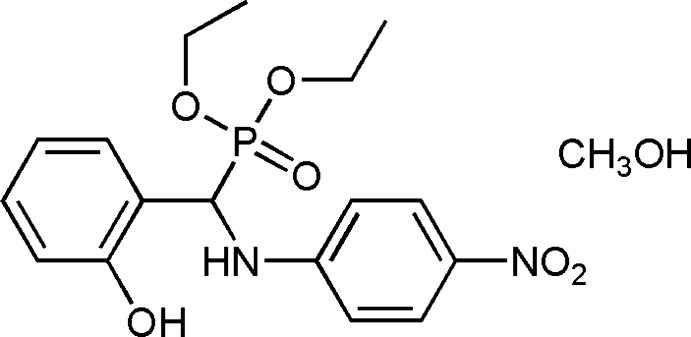


## Experimental   

### Crystal data  

C_17_H_21_N_2_O_6_P·CH_4_O
*M* *_r_* = 412.37
Triclinic, 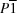

*a* = 9.401 (6) Å
*b* = 10.061 (6) Å
*c* = 11.963 (7) Å
α = 101.328 (10)°
β = 94.183 (10)°
γ = 104.549 (9)°
*V* = 1065.0 (12) Å^3^
*Z* = 2
Mo *K*α radiation
μ = 0.17 mm^−1^
*T* = 296 K
0.40 × 0.34 × 0.30 mm

### Data collection  

Bruker SMART 1K CCD area-detector diffractometer
Absorption correction: multi-scan (*SADABS*; Sheldrick, 2000 ▶) *T*_min_ = 0.935, *T*_max_ = 0.951
14569 measured reflections
5230 independent reflections
2934 reflections with *I* > 2σ(*I*)
*R*_int_ = 0.051

### Refinement  

*R*[*F* ^2^ > 2σ(*F* ^2^)] = 0.054
*wR*(*F* ^2^) = 0.138
*S* = 1.01
5230 reflections
262 parameters
H atoms treated by a mixture of independent and constrained refinement
Δρ_max_ = 0.31 e Å^−3^
Δρ_min_ = −0.27 e Å^−3^

### 

Data collection: *SMART* (Bruker, 2000 ▶); cell refinement: *SAINT* (Bruker, 2000 ▶); data reduction: *SAINT*; program(s) used to solve structure: *SHELXS97* (Sheldrick, 2008 ▶); program(s) used to refine structure: *SHELXL97* (Sheldrick, 2008 ▶); molecular graphics: *SHELXTL/PC* (Sheldrick, 2008 ▶); software used to prepare material for publication: *SHELXL97*.

## Supplementary Material

Crystal structure: contains datablock(s) I, New_Global_Publ_Block. DOI: 10.1107/S1600536814018649/pk2529sup1.cif

Structure factors: contains datablock(s) I. DOI: 10.1107/S1600536814018649/pk2529Isup2.hkl

Supporting information file. DOI: 10.1107/S1600536814018649/pk2529Isup3.cml

**Fig. 1.**
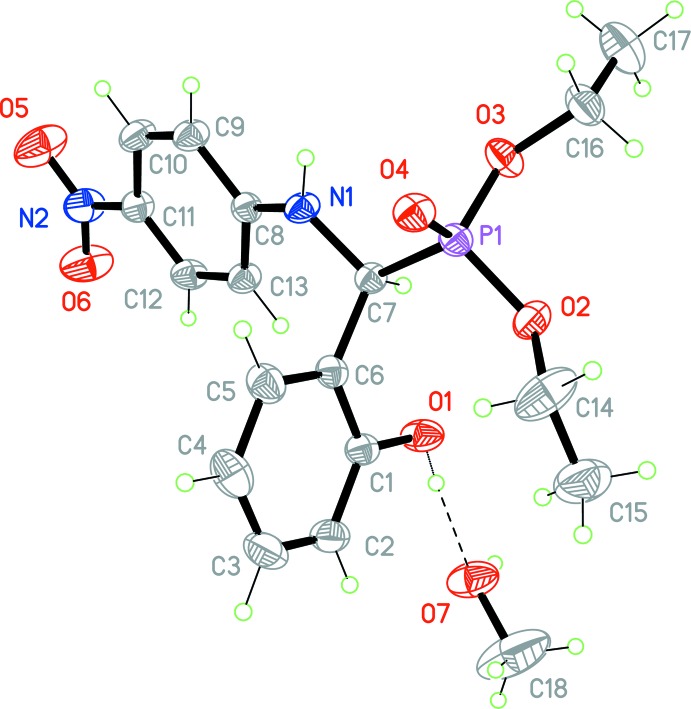
A view of the structure of the title compound with displacement ellipsoids drawn at the 30% probability level.

. DOI: 10.1107/S1600536814018649/pk2529fig1.tif
A view of the structure of the title compound with displacement ellipsoids drawn at the 30% probability level.

**Fig. 2.**
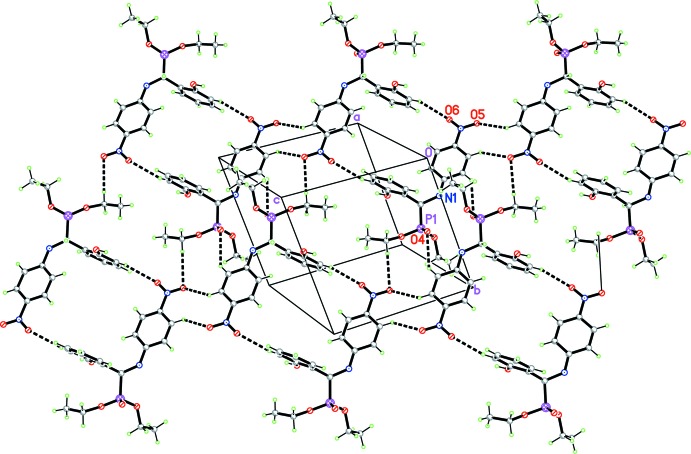
Crystal packing of the title compound, drawn so as to highlight the hydrogen-bonding interactions between molecules.

. DOI: 10.1107/S1600536814018649/pk2529fig2.tif
Crystal packing of the title compound, drawn so as to highlight the hydrogen-bonding inter­actions between mol­ecules.

CCDC reference: 1019639

Additional supporting information:  crystallographic information; 3D view; checkCIF report

## Figures and Tables

**Table 1:** Hydrogen-bond geometry (Å, °)

*D*—H⋯*A*	*D*—H	H⋯*A*	*D*⋯*A*	*D*—H⋯*A*
O7—H7*A*⋯O4^i^	0.82	2.00	2.819 (3)	172
O1—H1⋯O7	0.82	1.95	2.757 (3)	170
C10—H10⋯O5^ii^	0.93	2.53	3.308 (4)	141
N1—H1*A*⋯O4^iii^	0.82 (2)	2.14 (2)	2.959 (3)	171 (2)

Symmetry codes: (i) 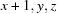; (ii) 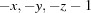; (iii) 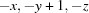.

## supplementary crystallographic information

### S1. Introduction

As mimics of natural amino acids, α-amino­phospho­nic acids and related derivatives are currently attracting a great deal of inter­est in medicinal chemistry due to their important biological effects (Arizpe, *et al.*, 2011). They have been reported to possess a wide range of biological functions. These include anti­bacterial activities (Allen *et al.*, 1978), action as inhibitors of enzymes such as rennin, HIV proteases, serine proteases and so on (Sieńczyk, *et al.*, 2009).

### S2.1. Synthesis and crystallization

The synthesis of o-cresol α-amino­phospho­nate N-derivatives with rigid structures was achieved through the Pudovik reaction reaction (Cherkasov *et al.*, 1998). We obtained the title compound following our earlier report (Wang *et al.*, 2012). The synthesis involved two steps: a) the Schiff bases were first prepared in a condensation of 4-nitro­aniline and salicyl­aldehyde in methanol solvent by refluxing equimolar amounts of reagents; b ) reaction of Schiff base with a di­ethyl phospho­nate in methanol solvent under reflux. The title compound was obtained from the filtrate after three days.

### S2.2. Refinement

The amine H atom was located in a difference Fourier map and refined freely. All other H atoms were placed in geometrically idealized positions and refined as riding, with C–H = 0.93-0.98 Å, O–H = 0.82 Å, and the Uiso(H) = 1.2Ueq (C) for benzene ring, C7, C14, C16 and Uiso(H) = 1.5Ueq (O, C) for O—H groups and C15, C17, C18.

### S3. Results and discussion

The crystal structure of the title compound is triclinic, with space group P1. As seen from Fig. 1, the P atom has tetra­hedral geometry involving two O-ethyl groups (O2, O3), one Cα atom (C7), and a double bond O atom (O4), which is the same as our earlier reports (Li *et al.*, 2008; Wang *et al.*, 2012). The C—P and P═O bond lengths are comparable to those in similar structures (Li *et al.* 2008; Wang *et al.*, 2012). Several hydrogen bonding inter­actions [O7—H7A···O4^i^ (i = x+1,y,z), O1—H1···O7, C10—H10···O5^ii^ (ii=-x, -y, -z-1), N1—H1A···O4^iii^ (iii=-x, -y+1, -z)] exist within in the crystal structure. The dihedral angle formed by the planes of the 4-nitro­aniline and 2-hy­droxy­phenyl groups is 84.08 (8)°.

### Crystal data

**Table:**

C_17_H_21_N_2_O_6_P·CH_4_O	*Z* = 2
*M**_r_* = 412.37	*F*(000) = 436
Triclinic, *P*1	*D*_x_ = 1.286 Mg m^−^^3^
Hall symbol: -P 1	Mo *K*α radiation, λ = 0.71073 Å
*a* = 9.401 (6) Å	Cell parameters from 1930 reflections
*b* = 10.061 (6) Å	θ = 1.8–28.3°
*c* = 11.963 (7) Å	µ = 0.17 mm^−^^1^
α = 101.328 (10)°	*T* = 296 K
β = 94.183 (10)°	Block, yellow
γ = 104.549 (9)°	0.4 × 0.34 × 0.3 mm
*V* = 1065.0 (12) Å^3^	

### Data collection

**Table:**

Bruker SMART 1K CCD area-detector diffractometer	5230 independent reflections
Radiation source: fine-focus sealed tube	2934 reflections with *I* > 2σ(*I*)
Graphite monochromator	*R*_int_ = 0.051
ω scans	θ_max_ = 28.2°, θ_min_ = 1.8°
Absorption correction: multi-scan (*SADABS*; Sheldrick, 2000)	*h* = −12→12
*T*_min_ = 0.935, *T*_max_ = 0.951	*k* = −13→13
14569 measured reflections	*l* = −15→15

### Refinement

**Table:**

Refinement on *F*^2^	Primary atom site location: structure-invariant direct methods
Least-squares matrix: full	Secondary atom site location: difference Fourier map
*R*[*F*^2^ > 2σ(*F*^2^)] = 0.054	Hydrogen site location: inferred from neighbouring sites
*wR*(*F*^2^) = 0.138	H atoms treated by a mixture of independent and constrained refinement
*S* = 1.01	*w* = 1/[σ^2^(*F*_o_^2^) + (0.0575*P*)^2^ + 0.0659*P*] where *P* = (*F*_o_^2^ + 2*F*_c_^2^)/3
5230 reflections	(Δ/σ)_max_ < 0.001
262 parameters	Δρ_max_ = 0.31 e Å^−^^3^
0 restraints	Δρ_min_ = −0.27 e Å^−^^3^

### Special details

**Table:**

Geometry. All e.s.d.'s (except the e.s.d. in the dihedral angle between two l.s. planes) are estimated using the full covariance matrix. The cell e.s.d.'s are taken into account individually in the estimation of e.s.d.'s in distances, angles and torsion angles; correlations between e.s.d.'s in cell parameters are only used when they are defined by crystal symmetry. An approximate (isotropic) treatment of cell e.s.d.'s is used for estimating e.s.d.'s involving l.s. planes.
Refinement. Refinement of *F*^2^ against ALL reflections. The weighted *R*-factor *wR* and goodness of fit *S* are based on *F*^2^, conventional *R*-factors *R* are based on *F*, with *F* set to zero for negative *F*^2^. The threshold expression of *F*^2^ > σ(*F*^2^) is used only for calculating *R*-factors(gt) *etc*. and is not relevant to the choice of reflections for refinement. *R*-factors based on *F*^2^ are statistically about twice as large as those based on *F*, and *R*- factors based on ALL data will be even larger.

### Fractional atomic coordinates and isotropic or equivalent isotropic displacement parameters (Å^2^)

**Table:**

	*x*	*y*	*z*	*U*_iso_*/*U*_eq_	
C1	0.4255 (3)	0.3224 (2)	0.17400 (19)	0.0408 (5)	
C2	0.4364 (3)	0.2362 (3)	0.2493 (2)	0.0538 (7)	
H2	0.5292	0.2347	0.2808	0.065*	
C3	0.3111 (4)	0.1532 (3)	0.2778 (2)	0.0655 (8)	
H3	0.3192	0.0962	0.3291	0.079*	
C4	0.1720 (4)	0.1538 (3)	0.2302 (3)	0.0692 (8)	
H4	0.0868	0.0979	0.2497	0.083*	
C5	0.1618 (3)	0.2385 (3)	0.1535 (2)	0.0538 (7)	
H5	0.0688	0.2379	0.1207	0.065*	
C6	0.2871 (3)	0.3241 (2)	0.12431 (18)	0.0378 (5)	
C7	0.2760 (2)	0.4232 (2)	0.04485 (17)	0.0348 (5)	
H7	0.3749	0.4597	0.0239	0.042*	
C8	0.2056 (2)	0.2631 (2)	−0.14895 (19)	0.0372 (5)	
C9	0.1083 (3)	0.2150 (2)	−0.2517 (2)	0.0467 (6)	
H9	0.0257	0.2492	−0.2595	0.056*	
C10	0.1327 (3)	0.1185 (3)	−0.3410 (2)	0.0526 (7)	
H10	0.0681	0.0884	−0.4092	0.063*	
C11	0.2548 (3)	0.0660 (2)	−0.3287 (2)	0.0449 (6)	
C12	0.3527 (3)	0.1124 (3)	−0.2294 (2)	0.0472 (6)	
H12	0.4347	0.0772	−0.2227	0.057*	
C13	0.3303 (3)	0.2105 (2)	−0.1397 (2)	0.0428 (6)	
H13	0.3976	0.2422	−0.0729	0.051*	
C14	0.3182 (4)	0.6256 (4)	0.3468 (2)	0.0941 (12)	
H14A	0.2782	0.7004	0.3841	0.113*	
H14B	0.2476	0.5363	0.3455	0.113*	
C15	0.4561 (4)	0.6334 (4)	0.4116 (3)	0.0989 (12)	
H15A	0.5020	0.5674	0.3700	0.148*	
H15B	0.4377	0.6111	0.4846	0.148*	
H15C	0.5206	0.7269	0.4237	0.148*	
C16	0.2262 (3)	0.8152 (3)	0.0724 (2)	0.0600 (7)	
H16A	0.2731	0.8615	0.1500	0.072*	
H16B	0.1208	0.8069	0.0696	0.072*	
C17	0.2894 (4)	0.8987 (3)	−0.0089 (3)	0.0853 (10)	
H17A	0.3945	0.9110	−0.0026	0.128*	
H17B	0.2697	0.9891	0.0087	0.128*	
H17C	0.2453	0.8505	−0.0858	0.128*	
C18	0.8676 (5)	0.4263 (6)	0.3596 (3)	0.154 (2)	
H18A	0.9379	0.3743	0.3716	0.232*	
H18B	0.7854	0.3987	0.4010	0.232*	
H18C	0.9143	0.5253	0.3868	0.232*	
N1	0.1748 (2)	0.3567 (2)	−0.06050 (16)	0.0415 (5)	
N2	0.2802 (3)	−0.0373 (3)	−0.4225 (2)	0.0650 (7)	
O1	0.54719 (18)	0.4078 (2)	0.14493 (15)	0.0566 (5)	
H1	0.6220	0.4018	0.1811	0.085*	
O2	0.33759 (18)	0.63846 (17)	0.22920 (13)	0.0518 (4)	
O3	0.25022 (19)	0.67641 (16)	0.04156 (14)	0.0525 (5)	
O4	0.06458 (17)	0.53339 (17)	0.14848 (13)	0.0478 (4)	
O5	0.1927 (3)	−0.0771 (3)	−0.51111 (19)	0.1029 (9)	
O6	0.3885 (3)	−0.0818 (2)	−0.41134 (17)	0.0919 (8)	
O7	0.8177 (2)	0.3987 (3)	0.24407 (17)	0.0785 (6)	
H7A	0.8843	0.4359	0.2104	0.118*	
P1	0.21847 (7)	0.57045 (6)	0.12230 (5)	0.03809 (18)	
H1A	0.107 (3)	0.391 (2)	−0.077 (2)	0.045 (7)*	

### Atomic displacement parameters (Å^2^)

**Table:**

	*U*^11^	*U*^22^	*U*^33^	*U*^12^	*U*^13^	*U*^23^
C1	0.0458 (14)	0.0433 (14)	0.0362 (12)	0.0202 (11)	0.0041 (11)	0.0060 (11)
C2	0.0633 (18)	0.0542 (16)	0.0482 (15)	0.0253 (14)	−0.0009 (13)	0.0122 (13)
C3	0.092 (2)	0.0552 (18)	0.0595 (18)	0.0284 (17)	0.0110 (17)	0.0260 (14)
C4	0.073 (2)	0.0569 (18)	0.081 (2)	0.0077 (16)	0.0271 (17)	0.0307 (16)
C5	0.0478 (16)	0.0494 (16)	0.0677 (18)	0.0133 (12)	0.0112 (13)	0.0202 (14)
C6	0.0427 (13)	0.0345 (12)	0.0383 (12)	0.0170 (10)	0.0074 (10)	0.0043 (10)
C7	0.0304 (12)	0.0384 (12)	0.0371 (12)	0.0127 (10)	0.0051 (10)	0.0069 (10)
C8	0.0386 (13)	0.0356 (12)	0.0384 (12)	0.0127 (10)	0.0057 (10)	0.0070 (10)
C9	0.0420 (14)	0.0532 (15)	0.0473 (14)	0.0244 (12)	−0.0002 (11)	0.0041 (12)
C10	0.0521 (16)	0.0589 (16)	0.0433 (14)	0.0201 (13)	−0.0072 (12)	0.0010 (12)
C11	0.0521 (15)	0.0439 (14)	0.0401 (13)	0.0229 (12)	0.0043 (11)	0.0008 (11)
C12	0.0491 (15)	0.0530 (15)	0.0478 (15)	0.0301 (12)	0.0070 (12)	0.0093 (12)
C13	0.0401 (13)	0.0476 (14)	0.0421 (13)	0.0196 (11)	0.0015 (11)	0.0047 (11)
C14	0.077 (2)	0.154 (4)	0.0440 (17)	0.040 (2)	0.0036 (17)	−0.005 (2)
C15	0.131 (3)	0.117 (3)	0.0546 (19)	0.058 (3)	−0.007 (2)	0.008 (2)
C16	0.0649 (18)	0.0428 (15)	0.0774 (19)	0.0221 (14)	0.0101 (15)	0.0153 (14)
C17	0.102 (3)	0.0560 (19)	0.106 (3)	0.0203 (18)	0.030 (2)	0.0333 (19)
C18	0.090 (3)	0.273 (6)	0.081 (3)	−0.008 (3)	−0.006 (2)	0.074 (4)
N1	0.0372 (11)	0.0475 (12)	0.0416 (11)	0.0229 (10)	−0.0020 (9)	0.0017 (9)
N2	0.0834 (18)	0.0676 (16)	0.0481 (14)	0.0422 (14)	−0.0002 (13)	−0.0021 (12)
O1	0.0382 (10)	0.0749 (13)	0.0640 (12)	0.0186 (9)	0.0022 (9)	0.0298 (10)
O2	0.0484 (10)	0.0560 (11)	0.0440 (10)	0.0116 (8)	−0.0047 (8)	0.0020 (8)
O3	0.0708 (12)	0.0400 (9)	0.0556 (11)	0.0256 (9)	0.0171 (9)	0.0143 (8)
O4	0.0395 (9)	0.0560 (10)	0.0531 (10)	0.0234 (8)	0.0092 (8)	0.0094 (8)
O5	0.1169 (19)	0.120 (2)	0.0634 (14)	0.0713 (16)	−0.0256 (14)	−0.0358 (13)
O6	0.1136 (18)	0.1124 (18)	0.0664 (14)	0.0860 (16)	−0.0014 (13)	−0.0079 (12)
O7	0.0485 (12)	0.1205 (19)	0.0717 (14)	0.0160 (12)	0.0039 (10)	0.0441 (13)
P1	0.0382 (3)	0.0382 (3)	0.0402 (3)	0.0168 (3)	0.0047 (3)	0.0059 (3)

### Geometric parameters (Å, º)

**Table:**

C1—O1	1.360 (3)	C14—C15	1.438 (4)
C1—C2	1.382 (3)	C14—O2	1.458 (3)
C1—C6	1.396 (3)	C14—H14A	0.9700
C2—C3	1.370 (4)	C14—H14B	0.9700
C2—H2	0.9300	C15—H15A	0.9600
C3—C4	1.390 (4)	C15—H15B	0.9600
C3—H3	0.9300	C15—H15C	0.9600
C4—C5	1.383 (4)	C16—O3	1.451 (3)
C4—H4	0.9300	C16—C17	1.469 (4)
C5—C6	1.386 (3)	C16—H16A	0.9700
C5—H5	0.9300	C16—H16B	0.9700
C6—C7	1.522 (3)	C17—H17A	0.9600
C7—N1	1.454 (3)	C17—H17B	0.9600
C7—P1	1.812 (2)	C17—H17C	0.9600
C7—H7	0.9800	C18—O7	1.377 (4)
C8—N1	1.371 (3)	C18—H18A	0.9600
C8—C9	1.401 (3)	C18—H18B	0.9600
C8—C13	1.407 (3)	C18—H18C	0.9600
C9—C10	1.371 (3)	N1—H1A	0.82 (2)
C9—H9	0.9300	N2—O6	1.219 (3)
C10—C11	1.389 (3)	N2—O5	1.225 (3)
C10—H10	0.9300	O1—H1	0.8200
C11—C12	1.372 (3)	O2—P1	1.5557 (18)
C11—N2	1.450 (3)	O3—P1	1.5638 (18)
C12—C13	1.376 (3)	O4—P1	1.4734 (18)
C12—H12	0.9300	O7—H7A	0.8200
C13—H13	0.9300		
			
O1—C1—C2	122.1 (2)	O2—C14—H14A	109.3
O1—C1—C6	117.4 (2)	C15—C14—H14B	109.3
C2—C1—C6	120.6 (2)	O2—C14—H14B	109.3
C3—C2—C1	120.4 (3)	H14A—C14—H14B	108.0
C3—C2—H2	119.8	C14—C15—H15A	109.5
C1—C2—H2	119.8	C14—C15—H15B	109.5
C2—C3—C4	120.2 (3)	H15A—C15—H15B	109.5
C2—C3—H3	119.9	C14—C15—H15C	109.5
C4—C3—H3	119.9	H15A—C15—H15C	109.5
C5—C4—C3	119.2 (3)	H15B—C15—H15C	109.5
C5—C4—H4	120.4	O3—C16—C17	109.0 (2)
C3—C4—H4	120.4	O3—C16—H16A	109.9
C4—C5—C6	121.5 (3)	C17—C16—H16A	109.9
C4—C5—H5	119.3	O3—C16—H16B	109.9
C6—C5—H5	119.3	C17—C16—H16B	109.9
C5—C6—C1	118.2 (2)	H16A—C16—H16B	108.3
C5—C6—C7	121.6 (2)	C16—C17—H17A	109.5
C1—C6—C7	120.2 (2)	C16—C17—H17B	109.5
N1—C7—C6	113.91 (19)	H17A—C17—H17B	109.5
N1—C7—P1	109.45 (15)	C16—C17—H17C	109.5
C6—C7—P1	108.85 (14)	H17A—C17—H17C	109.5
N1—C7—H7	108.2	H17B—C17—H17C	109.5
C6—C7—H7	108.2	O7—C18—H18A	109.5
P1—C7—H7	108.2	O7—C18—H18B	109.5
N1—C8—C9	119.2 (2)	H18A—C18—H18B	109.5
N1—C8—C13	122.4 (2)	O7—C18—H18C	109.5
C9—C8—C13	118.4 (2)	H18A—C18—H18C	109.5
C10—C9—C8	121.1 (2)	H18B—C18—H18C	109.5
C10—C9—H9	119.5	C8—N1—C7	123.21 (19)
C8—C9—H9	119.5	C8—N1—H1A	115.2 (17)
C9—C10—C11	119.4 (2)	C7—N1—H1A	119.4 (17)
C9—C10—H10	120.3	O6—N2—O5	121.8 (2)
C11—C10—H10	120.3	O6—N2—C11	119.1 (2)
C12—C11—C10	120.7 (2)	O5—N2—C11	119.1 (2)
C12—C11—N2	119.7 (2)	C1—O1—H1	109.5
C10—C11—N2	119.6 (2)	C14—O2—P1	125.58 (18)
C11—C12—C13	120.4 (2)	C16—O3—P1	121.48 (16)
C11—C12—H12	119.8	C18—O7—H7A	109.5
C13—C12—H12	119.8	O4—P1—O2	114.76 (10)
C12—C13—C8	120.0 (2)	O4—P1—O3	114.66 (10)
C12—C13—H13	120.0	O2—P1—O3	104.51 (10)
C8—C13—H13	120.0	O4—P1—C7	114.21 (10)
C15—C14—O2	111.4 (3)	O2—P1—C7	105.37 (10)
C15—C14—H14A	109.3	O3—P1—C7	101.94 (10)
			
O1—C1—C2—C3	179.0 (2)	C9—C8—C13—C12	−1.3 (3)
C6—C1—C2—C3	−1.2 (4)	C9—C8—N1—C7	−173.7 (2)
C1—C2—C3—C4	0.7 (4)	C13—C8—N1—C7	7.8 (3)
C2—C3—C4—C5	0.4 (4)	C6—C7—N1—C8	−72.5 (3)
C3—C4—C5—C6	−0.9 (4)	P1—C7—N1—C8	165.39 (18)
C4—C5—C6—C1	0.4 (4)	C12—C11—N2—O6	0.2 (4)
C4—C5—C6—C7	−176.7 (2)	C10—C11—N2—O6	179.3 (3)
O1—C1—C6—C5	−179.6 (2)	C12—C11—N2—O5	−179.4 (3)
C2—C1—C6—C5	0.6 (3)	C10—C11—N2—O5	−0.3 (4)
O1—C1—C6—C7	−2.4 (3)	C15—C14—O2—P1	150.2 (2)
C2—C1—C6—C7	177.8 (2)	C17—C16—O3—P1	170.2 (2)
C5—C6—C7—N1	−49.0 (3)	C14—O2—P1—O4	21.6 (3)
C1—C6—C7—N1	133.9 (2)	C14—O2—P1—O3	148.0 (2)
C5—C6—C7—P1	73.4 (2)	C14—O2—P1—C7	−104.9 (2)
C1—C6—C7—P1	−103.7 (2)	C16—O3—P1—O4	58.8 (2)
N1—C8—C9—C10	−178.1 (2)	C16—O3—P1—O2	−67.7 (2)
C13—C8—C9—C10	0.5 (4)	C16—O3—P1—C7	−177.30 (19)
C8—C9—C10—C11	0.9 (4)	N1—C7—P1—O4	56.04 (18)
C9—C10—C11—C12	−1.6 (4)	C6—C7—P1—O4	−69.04 (17)
C9—C10—C11—N2	179.3 (2)	N1—C7—P1—O2	−177.09 (14)
C10—C11—C12—C13	0.8 (4)	C6—C7—P1—O2	57.82 (17)
N2—C11—C12—C13	179.9 (2)	N1—C7—P1—O3	−68.20 (17)
C11—C12—C13—C8	0.6 (4)	C6—C7—P1—O3	166.71 (15)
N1—C8—C13—C12	177.3 (2)		

### Hydrogen-bond geometry (Å, º)

**Table:**

*D*—H···*A*	*D*—H	H···*A*	*D*···*A*	*D*—H···*A*
O7—H7*A*···O4^i^	0.82	2.00	2.819 (3)	172
O1—H1···O7	0.82	1.95	2.757 (3)	170
C10—H10···O5^ii^	0.93	2.53	3.308 (4)	141
N1—H1*A*···O4^iii^	0.82 (2)	2.14 (2)	2.959 (3)	171 (2)

Symmetry codes: (i) *x*+1, *y*, *z*; (ii) −*x*, −*y*, −*z*−1; (iii) −*x*, −*y*+1, −*z*.
