# Supplementary figures and images for: Crystal structure of diethyl [(4-nitro­phenyl­amino)(2-hy­droxy­phen­yl)meth­yl]phospho­nate methanol monosolvate
Source: Acta Crystallogr Sect E Struct Rep Online. 2014 Aug 30;70(Pt 9):o1053–4. doi: 10.1107/S1600536814018649 (PMC4186192; doi:10.1107/S1600536814018649)

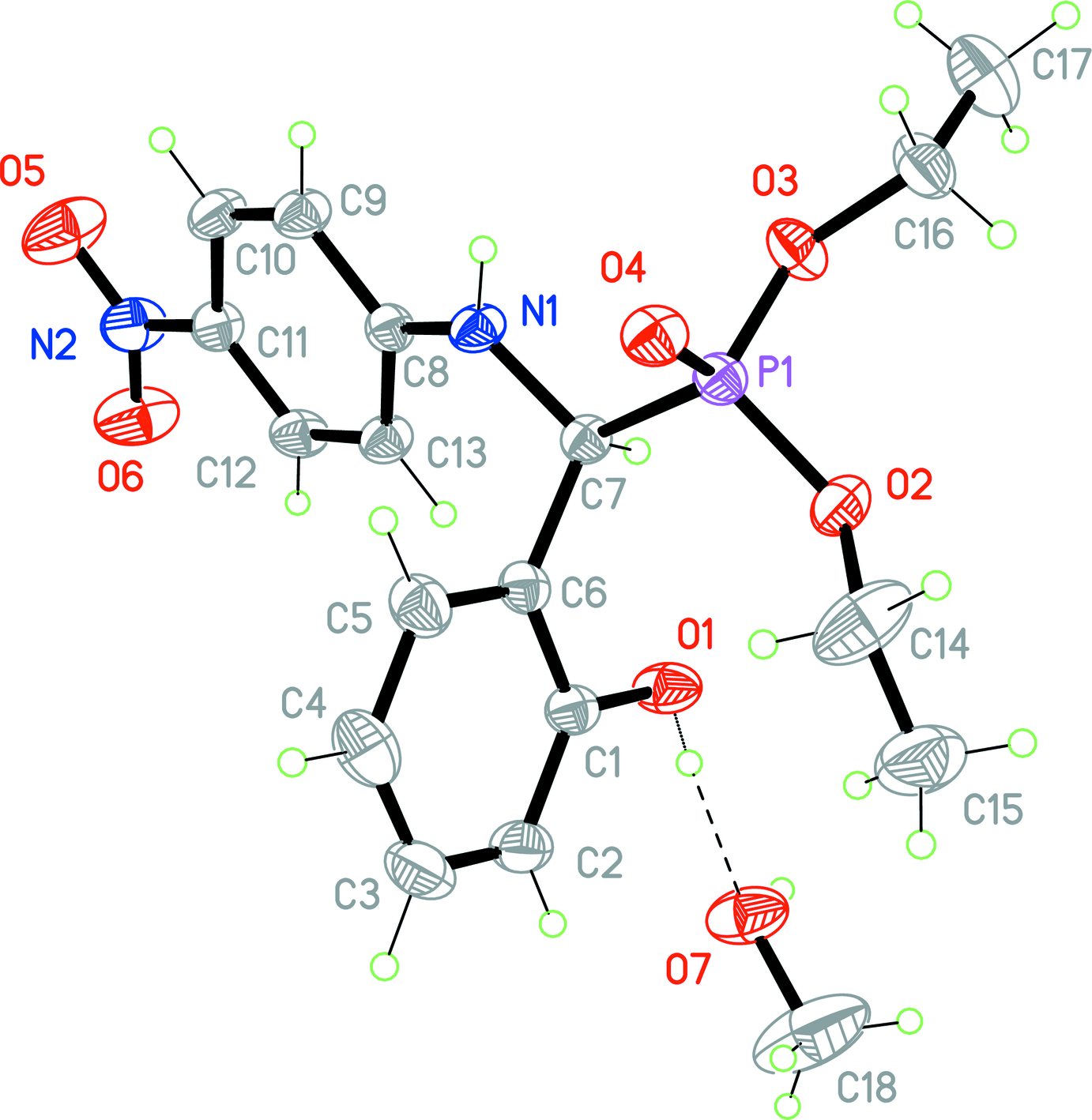

Supplement: Supplementary file 4 [file e-70-o1053-fig1.tif]

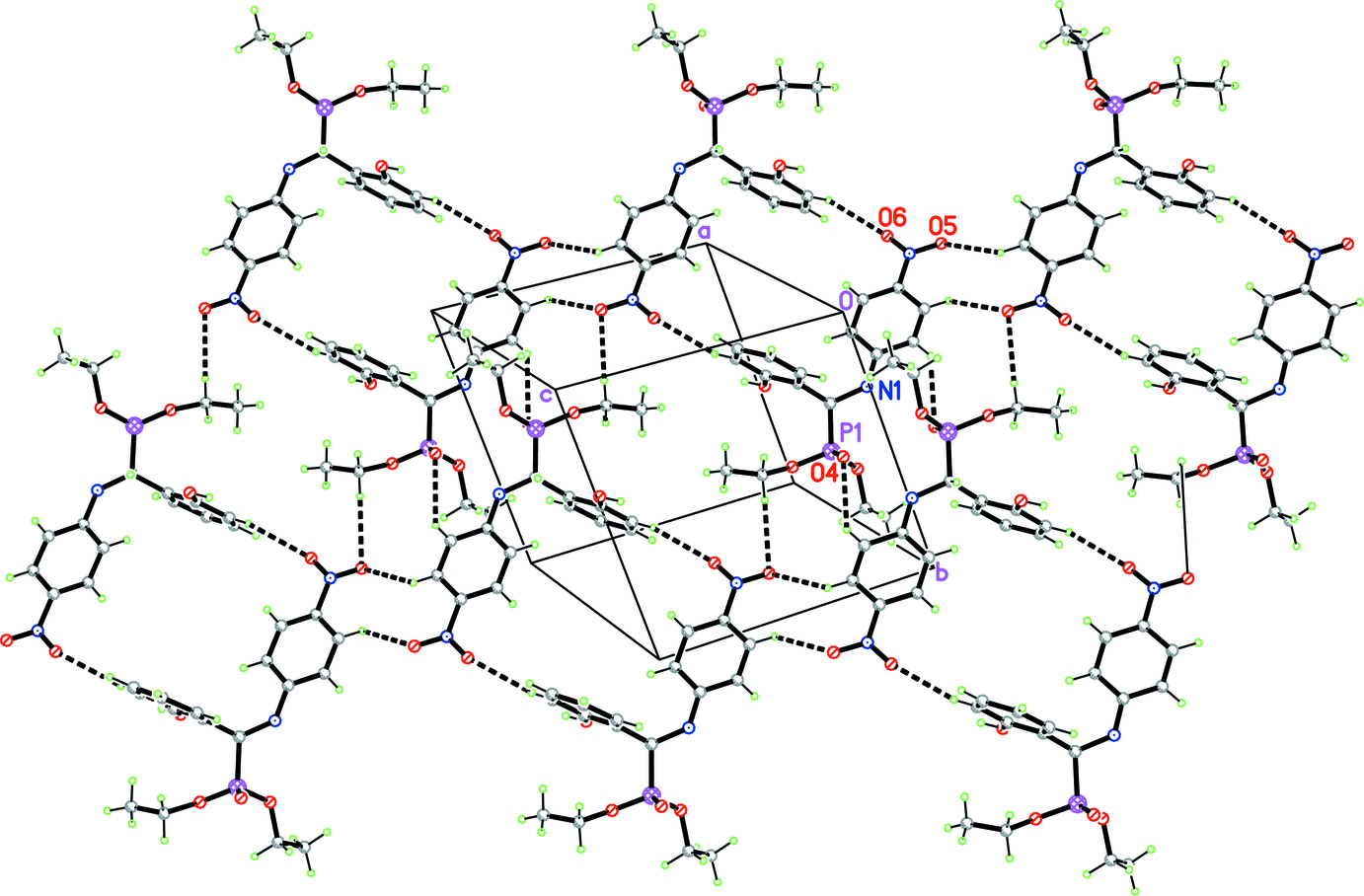

Supplement: Supplementary file 5 [file e-70-o1053-fig2.tif]
